# The Oxylipin Dependent Quorum Sensing System enhances *Pseudomonas aeruginosa* dissemination during burn-associated infection

**DOI:** 10.1371/journal.ppat.1013885

**Published:** 2026-01-20

**Authors:** Eriel Martínez, Hansol Im, Mohammed Mohasin, Landon Wilson, Javier Campos-Gomez, Carlos J. Orihuela

**Affiliations:** 1 Department of Microbiology, Heersink School of Medicine, The University of Alabama at Birmingham, Birmingham, Alabama, United States of America; 2 Department of Medicine, Division of Pulmonary, Allergy and Critical Medicine, The University of Alabama at Birmingham, Birmingham, Alabama, United States of America; 3 Cystic Fibrosis Research Center, The University of Alabama at Birmingham, Birmingham, Alabama, United States of America; Nanyang Technological University, SINGAPORE

## Abstract

*Pseudomonas aeruginosa* is a leading cause of life-threatening infections in burn patients, yet the molecular cues driving its hypervirulence remain poorly understood. Here, we identify the Oxylipin Dependent Quorum Sensing (ODS) system as a key regulator of *P. aeruginosa* pathogenicity in the burn wound environment. Using a murine burn model, we show that thermal injury significantly increases free oleic acid levels in skin, which *P. aeruginosa* converts into oxylipin autoinducers (10-HOME and 7,10-DiHOME) via OdsA and OdsB. These molecules activate the ODS regulon, promoting bacterial invasion of burned tissue and dissemination to internal organs. ODS-deficient mutants exhibited markedly reduced skin colonization, impaired translocation across endothelial barriers, and attenuated mortality compared to wild-type strains, confirming the role of ODS in hypervirulence. Importantly, immunization with recombinant OdsA or treatment with a small-molecule OdsA inhibitor significantly improved survival and reduced bacterial dissemination in burned mice. High-throughput screening identified AB012 as a potent OdsA inhibitor, which competitively binds the enzyme’s catalytic site and suppresses oxylipin synthesis, ODS gene expression, and biofilm formation without affecting bacterial growth. In vivo, AB012 reduced bacterial burden and systemic spread following burn injury. Collectively, these findings reveal that *P. aeruginosa* exploits host-derived oleic acid to activate ODS and enhance virulence, and they highlight OdsA as a promising target for therapeutic intervention to prevent sepsis in burn patients.

## Author summary

Burn injuries put patients at high risk for severe infections, and *Pseudomonas aeruginosa* is one of the most dangerous bacteria involved. These infections often spread quickly from the wound to internal organs, leading to life-threatening sepsis. We wanted to understand why *P. aeruginosa* becomes so aggressive in burn wounds. Our research shows that burn injuries release oleic acid, a fatty acid normally stored in tissues. The bacteria sense this molecule and use it to activate a special communication system called ODS, which changes their behavior and makes them more invasive. When ODS is active, the bacteria penetrate deeper into tissue and spread throughout the body. We also discovered that blocking ODS, either by vaccinating against one of its key enzymes or by using a small-molecule inhibitor, can protect against deadly infections in mice. These findings reveal a new way that *P. aeruginosa* exploits burn injuries and point to ODS as a promising target for future therapies to improve outcomes for burn patients.

## Introduction

*Pseudomonas aeruginosa* is able to cause acute and chronic infections as result of its ability to sense and rapidly adapt to new or altered environmental conditions [1,2]. However, our understanding of the host-specific molecular cues and physiological biomarkers, which are responsible for its transition between aggressive and persistent phenotypes remains incomplete [3,4]. Recently, we described a new mechanism that allows *P. aeruginosa* to recognize the host and activate a specific genetic program that strongly affects bacterial physiology [5]. This system, called the Oxylipin-Dependent quorum sensing System (ODS), employs host-derived oleic acid as the precursor for the enzymatic synthesis of oxylipins, specifically (10*S*)-Hydroxy-(8*E*)-octadecenoic acid (10-HOME) and (7*S*,10*S*)-hydroxy-(8*E*)-octadecenoic acid (7,10-DiHOME) [6]. In controlled *in vitro* settings, 7-HOME and 7,10-DiHOME generated by the ODS autoinducer synthases, OdsA and OdsB, accumulate in the extracellular milieu following the addition of oleic acid [7]. These molecules, in turn, are imported into the bacterial cytoplasm and induce the expression of the ODS regulon, which controls twitching motility and biofilm formation [5,8]. Our earlier work demonstrated the contribution of ODS to *P. aeruginosa* virulence in plants and insects [8]. However, a role for ODS during *P. aeruginosa* infection of mammals has not been previously reported.

Oleic acid is the predominant unsaturated fatty acid in human adipose tissue and a major constituent across various tissues [9,10]. While the majority of oleic acid in healthy tissues is esterified with glycerol forming triglycerides, studies have shown a dramatic increase in the amount of free oleic acid present in tissues and plasma of patients who have recently experienced severe burn injuries [11,12]*.* Notably, the pathogenesis of *P. aeruginosa* during infection of burn lesions, is considerably different from the less invasive yet persistent phenotype observed during other types of infection such as that occurring in excisional open wounds or in the lung of cystic fibrosis patients [13–15]. One key difference being that burn-related *P. aeruginosa* infections are marked by their rapid dissemination via the bloodstream to distant organs; a feature that contributes to the development of sepsis and worsens morbidity and mortality of burn patients worldwide [16]. Indeed burn-related infections, particularly those caused by *P. aeruginosa*, are the leading cause of death among individuals hospitalized for burn injury [17,18].

The underlying molecular drivers behind *P. aeruginosa’s* hypervirulence when in a burn injury setting remained up to this point unknown. Accordingly, we tested the hypothesis that the oleic acid released as result of burn injury potentiate ODS activation in *P. aeruginosa* and this drives its hypervirulence. Our findings shed light on how *P. aeruginosa* recognizes a unique burn-related cue to become hypervirulent, and offers a new therapeutic target with the potential to prevent aggressive *P. aeruginosa* infection and dissemination following major burn injury.

## Results

### Burn injury increases susceptibility to *P. aeruginosa*

**Fig 1:**
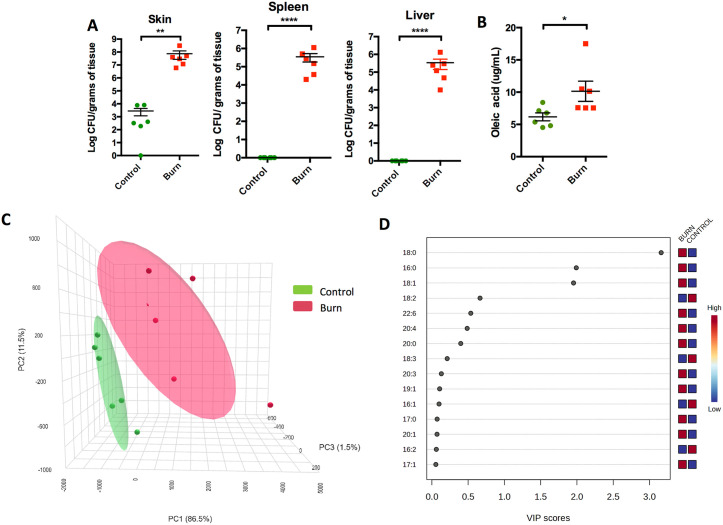
A mouse burn model reveals increased free fatty acid content at the burn wound site. A) Burned mice infected with PAO1 exhibited increased skin colonization compared to non-burned control, along with increased dissemination to both the spleen and liver. B) Burned skin showed a significant increase in free oleic acid concentration compared to non-burned controls. C) Three-dimensional Principal Component Analysis (3D-PCA) of mouse skin fatty acids (FAs) derived from high-resolution mass spectrometry reveals clear qualitative differences between Control and Burn groups, with distinct clustering and Components 1–3 accounting for 99.5% of the total variance. D) Variable Importance in Projection (VIP) analysis identifies FA 18:0, 18:1, and 16:0 as the major contributors driving group separation in this experimental model. Statistical analyses were performed using a nonparametric Mann-Whitney test. with asterisks denoting significance levels (* for P < 0.05, ** for P < 0.01, and **** for P < 0.0001). Six mice were utilized per experimental cohort. Data represent the combined results from at least two experiments, with each dot representing a biological replicate.

We first sought to validate the utility of the mouse model as means to investigate the molecular basis of burn-injury-induced susceptibility to *P. aeruginosa*. To do this, we used a previously described burn model [19]. Anesthetized mice received uniform third-degree burns by pressing a heated aluminum block against bare skin for 17 seconds, covering approximately 9% of total body surface area [20]. As immediately following the burn the skin remains intact, mice were in turn subcutaneously infected at the burn site with *P. aeruginosa* PAO1 using a tuberculin syringe. Consistent with the human condition, PAO1 robustly colonized burn wounds, whereas identically challenged non-burned mice were largely resistant to infection. Mean bacterial titers in excised tissues from burned mice were approximately 10,000 fold greater than those from control mice 48 hours after challenge (Fig 1A). All burn-injured mice infected with PAO1 also experienced bacterial dissemination to the spleen and liver. None of the unburned mice infected with PAO1 had this occur.

Our hypothesis portends that oleic acid levels are increased in burn tissues, and thereby more readily available to *P. aeruginosa* for conversion to oxylipins and activation of ODS. To test this, we excised skin from burned and control mice and examined the lipid composition using high-performance liquid chromatography-mass spectrometry. Most importantly, we observed almost a doubling in the amount of oleic acid that was detectable in excised tissue when previously burned (Fig 1B). What is more, we observed that the overall lipid profile was altered following burn injury as evidenced by differential clustering of samples in a PCA plot (Fig 1C). Further analyses indicated that differential levels of stearic acid (C18:0), palmitic acid (C16:0), and oleic acid (C18:1) in burned versus unburned samples were the primary drivers of this difference. Changes in the amount of linoleic acid (C18:2), docosahexaenoic acid (C22:6), arachidonic acid (C20:4), and arachidic acid (C20:0) also contributed albeit to less extent (Fig 1D).

### ODS promotes *P. aeruginosa* skin colonization and dissemination in a mouse burn model

**Fig 2:**
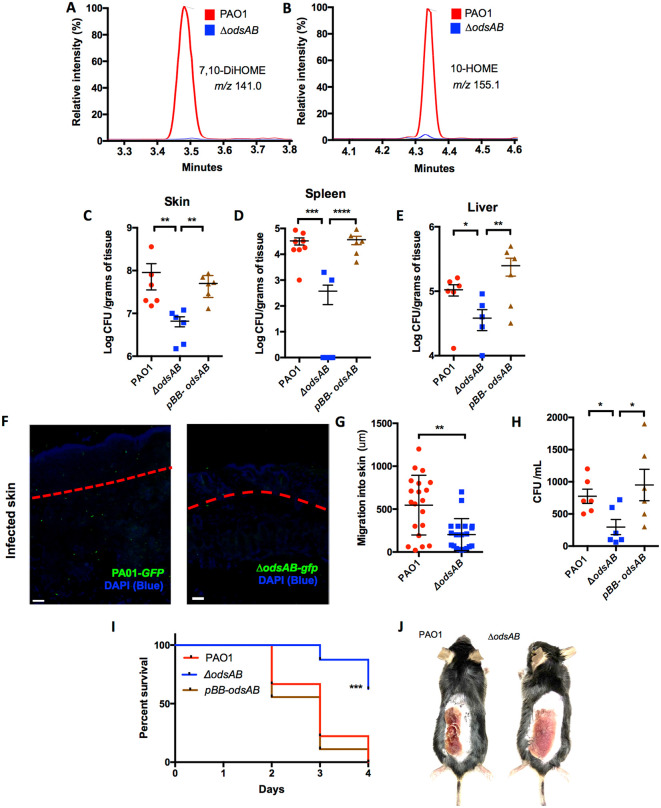
ODS promotes disseminated *P. aeruginosa* infection from burn wounds. LC/MS/MS spectrometry analysis of homogenized skin from burned mice infected with PAO1 or *ΔodsAB*. Reconstructed ion chromatograms demonstrate the presence of A) 7,10-DiHOME (m/z 297.3) and B) 10-HOME (m/z 155.1) exclusively in mice infected with PAO1 (depicted in red), while absent in those inoculated with *ΔodsAB* (depicted in blue). Additionally, burned mice infected with *ΔodsAB* exhibited C) reduced skin colonization compared to WT PAO1, along with impaired dissemination to both the D) spleen and E) liver. F) limited *ΔodsAB* burden within a cross-cut skin tissue section(Right) compared to WT PAO1 (Left). Whereas, G) Quantification of maximum penetration from captured images obtained from tissue sections confirmed deeper migration of PAO1 into the skin compared to *ΔodsAB.* Alongside this, results from H) an *in vitro* trans-well assay measuring the ability of an equal number of PAO1 to cross a confluent monolayer of MCEC in DMEM supplemented with 50 µM oleic acid. This assay revealed a significantly reduced translocation of the *ΔodsAB* mutant compared to the parental control strain. Notably, I) Kaplan Meier survival curves revealed a significant reduction in *ΔodsAB* infection-associated mortality compared to PAO1. J) burned mice infected with PAO1 displayed hindered skin healing relative to those infected with *ΔodsAB*, consistent with the greater bacterial burden. Representative image taken on day 3 post-infection. Likewise, Statistical analysis was performed using a nonparametric Mann-Whitney test with asterisks denoting significance levels (* for P < 0.05, ** for P < 0.01, and *** for P < 0.001). Mantel-Cox test was employed for survival data in Panel H. Six mice were utilized per experimental cohort, with exception to panel G, each dot denotes an individual mouse. Shown are the combined results from at least two experiments, with each dot representing a biological replicate.

Having shown that burn injury is associated with increased levels of oleic acid and greater bacterial burden in affected tissues, we next tested whether we could detect oxylipins in vivo, and in turn the requirement of OdsA and OdsB for this to occur along with hypervirulence of PAO1. Mass spectrometric analysis of burned skin samples only detected the ODS autoinducers 7,10-DiHOME and 10-HOME in samples from mice infected with wildtype PAO1 and not in those infected with *ΔodsAB* (Fig 2A and 2B respectively). Following burn injury, both PAO1 and *ΔodsAB* were able to colonize the injured skin. However, wild type-infected mice had approximately 10-fold more bacteria present in the skin than mice infected with *ΔodsAB.* What is more, this reduction in titer was abrogated when using a plasmid-bearing *odsAB*-complemented version of the mutant (*pBB-odsAB*; Fig 2C). Corroborating results were obtained following infection of burned mice with PAO1-*gfp* or *∆odsAB-gfp* and examination of skin sections under fluorescent microscopy. This revealed markedly lower numbers of the *∆odsAB* strain within burned tissue (S1A Fig). *ΔodsAB* also exhibited diminished dissemination from the skin to the spleen (Fig 2D) and the liver (Fig 2E), albeit to a lesser extent. Again, the *∆odsAB* mutant complemented in-trans with *pBB-odsAB* fully restored the wild-type phenotype (Fig 2D–E).

Microscopic examination of sectioned skin samples showed that *ΔodsAB-gfp* exhibited a reduced capacity to penetrate into the hypodermis layer when compared to PAO1*-gfp* (Fig 2F) and this difference was confirmed following quantitative analyses (Fig 2G). We sought to determine if this difference in invasion was due to bacterial quantity or an alteration in *P. aeruginosa* behavior as result of ODS activation. *In vitro* trans-well experiments testing the ability of an equal number of bacteria to cross a confluent monolayer of endothelial cells, demonstrated the requirement for both oleic acid and *odsAB* in efficient translocation (Figs 2H and S1B). Other aspects of infection were also affected by ODS activation, chief among these was altered mortality. While PAO1 parental strain resulted in 100% mortality within 48 hours post-burn/infection, *ΔodsAB* infected mice showed significantly higher survival, indicating a loss of virulence (Fig 2I). In parallel experiments having survivors, WT PAO1 infected mice had a delayed healing process compared to those infected with *ΔodsAB* (Fig 2J). These data establish an important role for ODS in *P. aeruginosa* virulence during burn wound infection in mammals, by influencing skin colonization and healing, dissemination to internal organs, and mortality. Along such lines, no differences in bacterial burden were observed between mice infected with WT and *ΔodsAB* strains in an unburned punch-biopsy excisional skin wound model (S1C Fig). Thus, the ODS system is of particular importance during burn wound infection.

### Immunized mice are protected from *P. aeruginosa* dissemination and death

**Fig 3:**
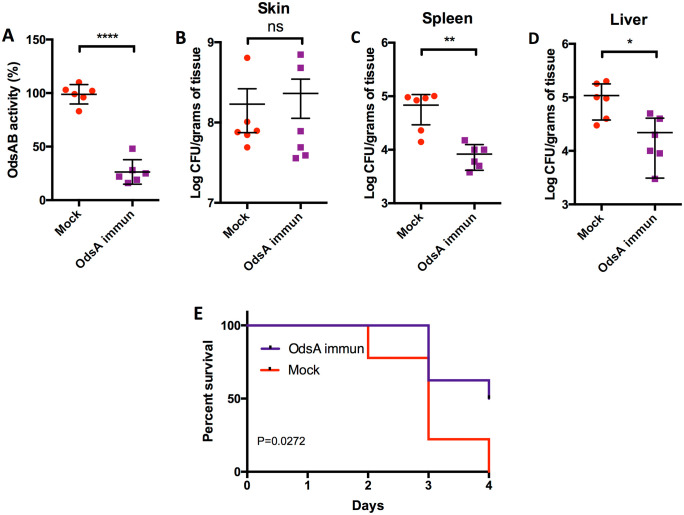
Immunized mice are protected from *P. aeruginosa* dissemination and mortality. A) Serum from mice immunized with recombinant OdsA, a 1:100 dilution, demonstrated the ability to inhibit oxylipin production by PAO1 *in vitro*, versus control sera, indicating that OdsA-specific antibodies can effectively block OdsA activity. B) No significant difference was observed in PAO1 levels of skin colonization between OdsA-immunized (OdsA Immun) and control mice (Mock). However, immunization with OdsA provided partial protection against bacterial dissemination from the skin to the C) spleen and D) liver as evidenced by lower bacterial counts. E) Kaplan-Meier survival curves depict the significant survival advantage of OdsA immunization. Non-immunized mice exhibited 100% mortality within four days post-infection with PAO1, whereas OdsA-immunized mice showed a 50% survival rate during the same timeframe. Statistical analyses were conducted using nonparametric Mann-Whitney test for panels A to D, with asterisks denoting significance levels (* for P < 0.05, ** for P < 0.01, and **** for P < 0.0001).while Mantel-Cox test was utilized for the survival data in panel E. Each cohort comprised six mice, and results are pooled from at least two independent experiments, with each dot representing a biological replicate.

OdsA is released by *P. aeruginosa* into the extracellular milieu, where it catalyzes the conversion of oleic acid into oxylipins [7]. This prompted us to explore whether antibody against OdsA could block its activity. Recombinant His-tagged OdsA was overexpressed and purified using an immobilized metal affinity chromatography column (S2A and S2B Fig). Mice were in turn immunized with recombinant OdsA, while a control group of mice received PBS. Upon completion of the immunization regimen, serum samples were assessed via immunoblotting to ascertain their ability to detect OdsA (S2C Fig). In vitro, sera from OdsA immunized mice effectively blocked the conversion of oleic acid to oxylipins (Fig 3A). To assess whether this conferred a protective effect, OdsA-immunized mice underwent burn injury and were infected with PAO1. Although no statistically significant difference was observed in terms of PAO1 skin colonization between the OdsA-immunized mice and PBS-vaccinated control group (Fig 3B), we observed reduced dissemination to the spleen (Fig 3C) and the liver (Fig 3D) compared to the control mice. Most importantly, a survival analysis revealed that OdsA immunization conferred significant protection against mortality in burned mice infected with PAO1 (Fig 3E). Finally, and supporting the specificity of our antibody to OdsA, both OdsA-immunized and non-immunized mice had unchanged levels of bacteria in the skin, spleen and liver following challenge with the *ΔodsAB* mutant (S3D–F Fig).

### An OdsA inhibitor reduces dissemination of *P. aeruginosa*

Encouraged by these immunization results, we developed a High-Throughput Screening (HTS) assay for testing whether OdsA activity was blocked. We aimed to identify small molecules targeting oxylipin synthesis for potential therapeutic treatment of *P. aeruginosa* infections in burn lesions. Our screening strategy relied on the differential solubility of oleic acid, the diol-synthase pathway substrate, compared to the oxylipin products. Notably, a 10 mM suspension of oleic acid, initially rendering a cloudy appearance, became clear when treated for one hour with a semi-purified fraction of the oxylipins synthases isolated from a free-cell supernatant of PAO1 (S3A Fig). This difference in transparency facilitated easy monitoring of the conversion of oleic acid into oxylipins by measuring the optical density of the surrounding medium at a wavelength of 600 nm (OD600). Compounds inhibiting oxylipin production were identified in wells where the medium remained at a high OD. Using this strategy, a proprietary in-house library of 200,624 compounds at Southern Research, Birmingham AL was screened at 10 µM.

The two most promising compounds internally labeled as AB01263211 (AB012) and AB00989558 (AB009) were selected for further validation in vitro, AB012 is an N-(pyridinyl) amide of a spiro-thiolane/cyclohexane system (S3B Fig), whereas AB009 is an N,N′-disubstituted urea bearing an α-methylbenzyl group on one nitrogen and a propargyl group on the other (S3C Fig). A 10-point dose-response assay determined its IC₅₀ value to be 11.34 and 6.92 µM, respectively. The compounds underwent retesting for inhibition of oxylipin synthesis *in vitro* utilizing the semi-purified fraction of oxylipin synthase enzymes employed during the initial screening process (S3D Fig).

**Fig 4:**
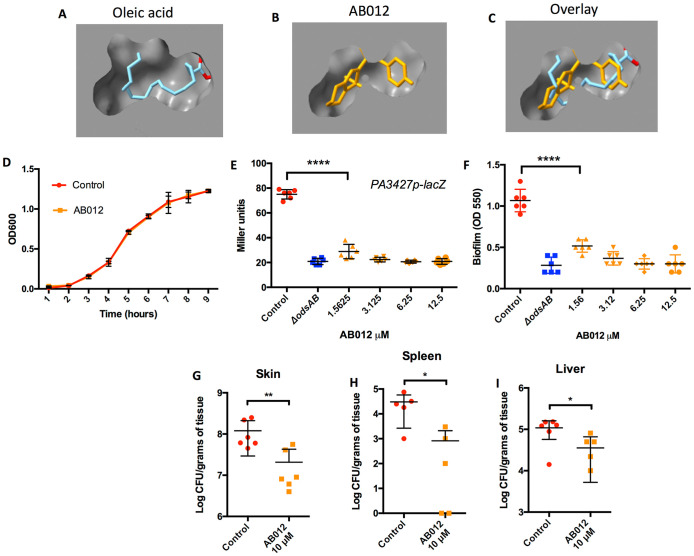
An OdsA inhibitor prevents dissemination of *P. aeruginosa.* A) Best docking poses of A) oleic acid and B) AB012 with OdsA. C) Superimposed structures showing AB012 and oleic acid occupy the same active site on OdsA. D) Growth curve of PAO1 treated with 10 µM AB012 shows no significant growth inhibition. E) AB012 also exhibited inhibition of lacZ expression under the control of the PA3427 promoter. PA3427 served as a reporter of ODS induction due to its high induction by oxylipins. F) AB012 inhibited *P. aeruginosa* biofilm formation in a concentration-dependent manner. G) In a burn wound infection model, mice treated intradermally with AB012 demonstrated reduced skin colonization, along with decreased dissemination to H) the spleen and I) the liver. Each dot denotes an individual mouse. Statistical analyses were conducted using nonparametric Mann-Whitney test, with asterisks denoting significance levels (* for P < 0.05, and **** for P < 0.0001). Six mice were utilized per experimental cohort. Shown are the combined results from at least two experiments, with each dot representing a biological replicate.

Using computational software, we docked AB012 and AB009, along with oleic acid, the natural ligand of OdsA to the predicted structure of OdsA as generated by AlphaFold. The binding energy profiles and molecular interactions of each ligand with the OdsA target are illustrated in Figs 4A–C and S4A. The best docking poses of AB012 and oleic acid predicted that both ligands bind within the same pocket, the putative active site (Fig 4C). The calculated predicted binding affinities of AB012 and oleic acid to OdsA were -7.793 and -6.679 kcal/mol, respectively (S1 Table). The inhibitory constant (Ki) for AB012 was approximately six-fold lower than that of oleic acid (1.94 μM vs. 12.71 μM), indicating higher binding potency. Superimposed docking images and analysis of intermolecular interactions support the conclusion that AB012 acts as a competitive inhibitor of OdsA, mimicking oleic acid. Both ligands were predicted as having strong hydrophobic interactions (distance <4.0 Å) with the same key residues: Phe260, Trp272, Ile545, and Tyr551 (S4B Fig). In contrast, the best docking pose of AB009 and its molecular interaction profile suggest that AB009 functions as an allosteric, non-competitive inhibitor. It is predicted to form stable interactions (distance <4.0 Å) with six residues located near the distal heme cofactor of OdsA, suggesting interference with a potential electron transfer mechanism critical to enzymatic activity (S4B Fig and S1 Video).

It is noteworthy, that AB012 did not affect the growth of *P. aeruginosa* PAO1 at 10 µM (Fig 4D); however, it significantly inhibited the expression of the ODS reporter gene *PA3427*, as demonstrated using a p*PA3427*-*lacZ* fusion reporter strain (Fig 4E). This was confirmed by RT-qPCR analysis, which showed a 43% reduction in *PA3427* gene expression in PAO1 cultured with oleic acid and treated with AB012, compared to control bacteria grown with oleic acid alone (n = 3, P < 0.05). In addition, AB012 exhibited a concentration-dependent inhibition of biofilm formation in PAO1, a trait previously attributed to ODS when in the presence of oleic acid, while it had no effect on *ΔodsAB* (Fig 4F). Finally, burned mice infected with PAO1 were administered 10 µM of AB012 intradermally immediately after challenge. Encouragingly, AB012-treated mice exhibited reduced PAO1 colonization of the skin (Fig 4G) and reduced dissemination to the spleen and liver compared to the untreated mice (Fig 4H and 4I). While further optimization is necessary, these proof-of-concept data support an important role for OdsA during burn-infection and that OdsA as a viable therapeutic target.

## Discussion

*P. aeruginosa* exhibits a remarkable capacity to initiate both acute and chronic infections, a trait attributed to its proficiency in perceiving and adjusting to dynamic host environments [1,2]. Although many studies have addressed the adaptive mechanisms facilitating *P. aeruginosa* pathogenicity in burn wounds [21–23], the specific signals governing the genetic programs leading to this type of acute infection remain largely unexplored. This study was motivated by the observation that *P. aeruginosa* infections of burn wounds frequently manifest heightened invasiveness and severity. Our goal was to unravel the molecular underpinnings of *P. aeruginosa* hyper-virulence during burn-related infections, focusing on the role of oleic acid, a major component of human adipose tissue, released during burn injury and the quorum sensing system ODS, which relies on oleic acid for production of its autoinducer.

Building upon previous findings obtained using a *Drosophila* model of infection [8], we sought to elucidate the involvement of the ODS regulatory system in virulence using a mouse burn model. Our findings with mice affirmed that burn injury drastically enhances susceptibility to infection, and that oleic acid is released as result of burn injury, but also showed that the latter is part of an altered lipid profile within the injured skin. Notably, oleic acid has been shown to have strong antimicrobial properties against Gram-positive bacteria such as *S. aureus* [24], a feature which suggests its release is actually a defense response that is co-opted by *P. aeruginosa*. Our detection of both 7-HOME and 7,10-DiHOME within burned tissues infected by *P. aeruginosa* and not in tissues infected with the OdsAB deficient mutant, indicate that indeed *P. aeruginosa* uses host-derived oleic acid to generate these autoinducers. Significantly, ODS activation not only promoted skin colonization, but also facilitated bacterial translocation to deep tissue causing dissemination to internal organs, a hallmark of severe infections. The notion that the burn microenvironment is conducive to ODS activation was substantiated by reduction in bacterial burden in mice infected with the ODS-deficient mutant, as well the reduction seen in mice infected with wild type bacteria that were either immunized against OdsA or had been treated with small molecules that blocked OdsA activity. It is noteworthy that oleic acid typically is absent in the airway or lungs, as its presence can led to injury and inflammation [25]. This potentially explains why *P. aeruginosa* infections in context of cystic fibrosis or chronic airway diseases show less aggressiveness than in burn patients.

Our investigations unveiled a compelling distinction between the WT strain and the *ΔodsAB* mutant, with the latter exhibiting a significantly reduced capacity to migrate into the lower layers of skin. Our experiments with confluent endothelial cell monolayers performed *in vitro* indicate this was not simply the result of differences in bacterial burden, albeit this is likely a contributing factor in vivo. Along such lines, our choice of testing for differences on vascular endothelial cell layers is particularly relevant, considering that *P. aeruginosa* must traverse this specific cell type to access blood vessels. Previous reports from our research highlighted the inhibitory effect of ODS on flagella-dependent motility (swimming and swarming) while concurrently inducing Type-4 pili-dependent motility, known as twitching. Based on our most recent results, we now hypothesize that this specific ODS-dependent phenotype may facilitate adhesion to biotic surfaces, promoting subsequent internalization into host tissues. Consistent with this hypothesis, existing research underscores the importance of Type IV pili in *P. aeruginosa’s* adherence to epithelial cells. Moreover, there is supporting evidence indicating the involvement of twitching motility in the translocation of corneal epithelial cell multilayers by *P. aeruginosa*, demonstrated both *in vitro* and *in vivo* [26]. While our experiments do not pinpoint the precise mechanism through which ODS facilitates cell layer translocation, it unequivocally emphasizes the significance of this system in promoting the dissemination of *P. aeruginosa* across host organs. Finally, we cannot overlook the potential direct effect of oleic acid on other aspects of *P. aeruginosa* physiology or on ODS-derived oxylipins on host defenses, potentially aiding in *P. aeruginosa* dissemination. Recent findings have reported the presence of ODS-derived oxylipin, 10-HOME, in women with infected breast implants. In this instance, 10-HOME was shown to polarize CD4 + T cells to the Th1 subtype *in vitro* and in mice, suggesting a possible immunomodulatory role in facilitating *P. aeruginosa* dissemination [27].

We used a static crystal-violet biofilm assay to assess the impact of blocking OdsA activity on *P. aeruginosa* virulence potential. Although this assay is widely employed for initial characterization of bacterial biofilm formation ability, we concede that it does not fully recapitulate the complex structural and physiological features of biofilms formed in vivo. Static assays lack key environmental parameters such as shear flow, nutrient gradients, microoxic niches, and host-derived factors that shape the architecture and function of *P. aeruginosa* biofilms in wound tissues. Thus, while this assay provides a useful first approximation of ODS-dependent biofilm behavior, future studies using more physiologically relevant systems, such as flow-cell or microfluidic platforms, ex vivo skin or wound explants, and imaging modalities capable of resolving internal biofilm architecture, will be essential to define how ODS influences biofilm formation and dispersal under burn-like conditions.

To our knowledge, no other di-heme containing fatty acid oxygenase such as OdsA has been described in higher eukaryotes suggesting a lower risk of off-target binding of either antibody or AB012 in vivo. Encouragingly, our results demonstrated that a polyclonal antibody targeting OdsA effectively blocks ODS activity *in vitro*, and that mice immunized with OdsA showed partial protection against disseminated infection following burn injury. Our ongoing efforts will focus on the development of a monoclonal anti-OdsA for potential therapeutic interventions. Our findings also prompted us to develop a strategy to identify small molecules able to block the ODS pathway. Drugs targeting enzymes involved in oxylipin production in mammals and fungi, such as aspirin, diclofenac, ibuprofen, and imidazole derivatives, have been extensively commercialized. However, the role of oxylipins in bacterial pathogenesis has received less attention, and currently, there are no commercially available drugs specifically designed to block bacterial oxylipin synthesis. The HTS assay identified several promising candidates, two of which effectively inhibited oxylipin synthesis in vitro. Molecular docking studies with OdsA suggest that AB012 competes with oleic acid for the catalytic site, whereas AB009 inhibits OdsA activity via an allosteric mechanism. As a proof of concept for targeting OdsA in vivo, we selected AB012 due to its high predicted affinity and specific binding to the enzyme’s catalytic site. In our burn mouse model, AB012 significantly decreased *P. aeruginosa* dissemination to internal organs, presumably by inhibiting OdsA activity in vivo. While further expansion of the hit library is warranted, these findings highlight AB012’s therapeutic potential and support the ODS system as a viable target for intervention in burn-associated infections.

In summary, our study significantly advances our comprehension of the nuanced interplay between environmental cues and virulence in *P. aeruginosa*. We establish free oleic acid as a discernible burn marker recognized by *P. aeruginosa* through the ODS system, triggering a cascade of events that promote virulence. This study uncovers a role for oxylipins, produced by prokaryotes, akin to those seen in other pathogens like fungi, as pivotal signaling molecules in bacterial interactions with mammalian hosts [28]. Specifically, these findings shed light on the pathogenicity of *P. aeruginosa*, particularly in the context of heightened virulence during burn wound infections. Moreover, our research highlights a promising avenue for therapeutic intervention aimed at mitigating dissemination following infection.

## Methods

### Ethics statement

All animal experiments were reviewed and approved by the Institutional Animal Care and Use Committee at The University of Alabama at Birmingham, UAB (protocol no. IACUC-22197).

### Strains

*Pseudomonas aeruginosa* strain PAO1, sourced from the Manoil Lab at the University of Washington in Seattle, WA, USA, served as the parental strain throughout our investigation. The isogenic mutant *ΔodsAB* (diol synthase operon deletion mutant) was obtained following previously established protocols [8]. To complement the *ΔodsAB* mutant, the *odsAB* operon from the parental PAO1 strain was PCR-amplified and cloned into the pBBR1MCS vector using SphI–KpnI restriction sites (*pBB-odsAB*). Green fluorescent *P. aeruginosa* strains were generated through transformation with plasmids pMF230, which constitutively express GFP. Plasmids pMF230 (Addgene plasmids #62546), generously provided by Michael Franklin of Montana State University, were utilized for this purpose. *Escherichia coli* DH5α (Invitrogen) served as the host for plasmid constructions, while *E. coli* S17-1 λpir, a gift from Jorge Benitez of Morehouse School of Medicine, was utilized as a donor strain for bacterial conjugation when necessary. For bioluminescence measurements, *E. coli* DH5α harboring the pRPL3 plasmid was used as a nitric oxide reporter strain ^44^. Cultures were grown in LB medium to an OD_600_ of 0.5 and subsequently mixed with either the *ΔodsAB* mutant or its parental Pseudomonas aeruginosa PAO1 strain. Bioluminescence was recorded using a SPARK Multimode Microplate Reader (TECAN).

### Culture conditions

The strains were routinely cultivated in lysogeny broth (LB) medium at 30°C, with agar incorporated when solid medium was necessary. For biofilm formation assays, M63 medium was utilized, supplemented with 2% glucose, 5% casamino acids, and 1 mM MgSO_4_ (referred to as M63 complete). Antibiotics were supplemented as needed, with ampicillin (Amp) at 100 μg ml^−1^ and carbenicillin (Cb) at 300 μg ml^−1^. To induce oxylipin production and purification, cultures were supplemented with 90% oleic acid (Sigma 364525). When investigating biofilm formation *in vitro*, M63 complete media was supplemented with either 99% oleic acid (Sigma O1008) or purified oxylipins as required.

### Thin layer chromatography

Thin layer chromatography (TLC) experiments were conducted using Whatman silica gel plates (60 Å), measuring 20 × 10 cm with a thickness of 200 μm. The mobile phase consisted of a mixture of hexane:ether:acetic acid in proportions of 80:20:5, respectively. Visualization of the separated compounds on the TLC plates was achieved by treating them with a solution of 10% phosphomolybdic acid in ethanol.

### Purification of 10-HOME and 7,10-DiHOME oxylipins

The supernatant from a 500 ml PAO1 culture cultivated in M63 complete medium supplemented with 1% oleic acid was utilized for the purification of oxylipins produced through diol synthase activity. Following centrifugation of the culture at 8000 x g for 15 minutes, the supernatant was carefully retrieved and acidified to pH 2 using hydrochloric acid. Subsequently, a one-to-one volume ratio organic extraction was conducted employing ethyl acetate. The organic phase was then evaporated, yielding a dried mixture that was dissolved in 3 ml of ethyl acetate for further purification steps. Purification of the oxylipins was carried out utilizing an Isco Teledyne Combiflash Rf 200 equipped with four channels and a 340CF ELSD (evaporative light scattering detector). Pre-packed cartridges of Universal RediSep solid sample loading (5.0 g silica) were employed for crude product absorption, followed by purification on 24 g silica RediSep Rf Gold Silica columns (20–40 μm spherical silica) using an ascending gradient of ethyl acetate (solvent B) against hexane (solvent A). Fractions corresponding to each detected peak were pooled and evaporated before being dissolved in ethanol. The purity of the oxylipins was assessed through HPLC/MS analysis.

### Lipid extraction from mouse skin

A 100uL aliquot of bead homogenized mouse skin samples were added to 100uL of ice-cold PBS. A liquid-liquid extraction procedure using 750**μ**L mixture of Methanol:Chloroform (2:1) was added, then 250**μ**L of chloroform, followed by a 250**μ**L additional of *dd*H_2_0 (Bligh-Dyer Protocol) to each sample. Samples were then centrifuged at 3000xg for 10 minutes at 4^o^C to help facilitate the separation of phases of the samples. The bottom organic layer containing the lipids was then transferred to a new glass test tube and dried under nitrogen gas. Each sample was then re-suspended in 5000**μ**L of (2:1:1) 2-Propanol:Acetontrile:*dd*H_2_0 for mass spectrometry analyses.

### LC-MS/MS analysis (untargeted lipidomics)

An aliquot (2**μ**L) of each sample was loaded onto a Kinetex 2.1 x 100mm, 2.6μm F5, 100 Å reverse-phase column (Phenomenex, Torrance, CA). A gradient of 15–30% mobile phase B for 2 min, 30–48% B until 0.5 min, 48–82% for 8.5 min, 88–99% for 0.15min, hold for 0.75 min then re-equilibration at initial conditions for 3 minutes using an Exion UHPLC (Sciex, Toronto, Ontario) and a flow rate of 400**μ**l/min. The column temperature was set at 40^o^C to aid in lipid separation. The mobile phases are A) 50% ddH_2_O/30% Acetonitrile/20% 2-Propanol with 5mM Ammonium acetate and B) 90% Isopropyl alcohol/9%acetonitrile/1% ddH_2_O with 5mM Ammonium acetate respectively. The SCIEX 7600 + ZenoTof mass spectrometer (SCIEX, Toronto, Canada) was used to analyze the metabolite profile. The IonSpray voltages for negative mode were - 4500 V and the declustering potential was + /- 80 V. Ionspray GS1/GS2 and curtain gases were set at 50 psi, 70 psi and 35 psi respectively. The interface heater temperature was 600^o^C. Eluted compounds were subjected to a time-of-flight survey scan from *m/z* 200–1500 and the top 50 analytes were subjected to Data-dependent scans for MSMS analyses. Product ion time-of-flight scans to obtain the tandem mass spectra of the selected parent ion(s) over the range from m/z 70–1500 were collected over 10 msec intervals using a collision energy of -35 eV with a 20000 cps intensity threshold to activate the ZenoTrap function to improve lower abundant fragmentation spectra.Spectra were centroided and de-isotoped by Analyst OS software, version 3.4 (Sciex, Toronto, Canada).

### Data analysis and oleate quantification

LC-MS data were processed using MS-Dial vs 5.4.241021 (RIKEN Center, Yokohama City, Kanagawa) to identify peaks occurring across all samples, the peak areas and their retention times as well as possibly lipid annotations using the Lipid Library algorithm within the program. Verification, integration and quantification of Oleate was carried out by evaluating fragmentation spectra of each target using PeakView 2.2 software (SCIEX, Toronto, Ontario) against a standard curve of oleate from 100**μ**g/mL to 100ng/mL.

### HPLC/MS analysis of oxylipins

Mass spectrometry analysis was performed as described previously [29]. Purified 7,10 Di-HOME and 10-HOME were prepared as stock solutions at a concentration of 1 mg ml − 1 in ethanol. From these stock solutions, samples for analysis were prepared by diluting in ddH_2_O containing 0.1% formic acid. Each sample, with a 20 μl injection volume, was loaded onto a Synergi Hydro-RP 80A 250 × 2 mm C18 column (Phenomenex), employing a Shimadzu Prominence System Binary Pump (Shimadzu Scientific Instruments, Inc., Columbia, MD, USA) at a flow rate of 350 μl min − 1. Mobile phase A consisted of ddH_2_O with 0.1% formic acid, while mobile phase B comprised acetonitrile with 0.1% formic acid. The gradient elution started at 10% B and increased to 80% B over 11 min, followed by a ramp to 100% B at 14 min, then re-equilibrated to initial conditions over 6 min, resulting in a total runtime of 20 min per analysis. The SCIEX 4000 Triple Quadrupole Mass Spectrometer (Concord, Ontario, Canada) operated in ESI negative ion mode, with nitrogen serving as the nebulizer and curtain gas (CUR = 20). Collision gas, collision energy, and temperature were set at 10°C (−30 eV for 10-HOME, −34 eV for 7,10-DiHOME) and 600°C, respectively. Gas settings GS1 and GS2 were maintained at 40°C and 60°C, respectively. Analyst 1.6.2 software controlled the LC-MS/MS system.

### ODS activity assay

The ODS assay utilized for the HTS assay focused on the differential solubility properties of the diol-synthase pathway substrate, OA, compared to its oxylipin products. A suspension of OA in PBS at a concentration of 1 mg/mL results in a cloudy solution due to the formation of micelles. Upon treatment with semipurified diol-synthase enzymes, at protein concentration of 1mg/ml, the suspension becomes transparent, indicating the conversion of OA to more soluble oxylipins. To identify inhibitors of diol-synthase activity, compounds were tested for their ability to maintain the cloudiness of the OA suspension, as this would suggest inhibition of the enzyme. The cloudiness of the suspension was quantified by measuring its optical density at a wavelength of 600 nm (OD600). A clear suspension after treatment would indicate enzyme activity, while a cloudy suspension would signal inhibition. The assay was validated in a 384-well plate format and subsequently adapted for robotic automation. The same protocol was used to test the neutralizing activity of sera obtained from mice immunized with OdsA. In this variation, the conversion of oleic acid to oxylipins was monitored by thin-layer chromatography (TLC), and the resulting bands were quantified by densitometric analysis to determine the extent of enzyme inhibition.

### Quantitative real-time PCR (RT-qPCR) analysis

Total RNA was extracted from control and treated bacterial cultures using the RNeasy Plus Mini Kit (Qiagene) according to the manufacturer’s instructions. RNA concentration and purity were determined using a NanoDrop spectrophotometer. cDNA synthesis was performed using High-Capacity cDNA Reverse Transcription Kit (Applied Biosystem) with 500 ng of total RNA in a 20 µL reaction volume. Quantitative real-time PCR was carried out using Fisherbrand SYBR Green qPCR Master Mix on a CFX Opus 96 system (Bio-Rad). Each 20 µL qPCR reaction contained 10 µL of SYBR Green Master Mix, 0.4 µM of each primer, and 1 µL of cDNA template. The thermal cycling conditions were as follows: initial denaturation at 95°C for 2 minutes, followed by 40 cycles of 95°C for 15 seconds and 60°C for 1 minute. Melting curve analysis was performed to verify the specificity of amplification. Gene expression of the target gene PA3427 was normalized to the expression of the housekeeping gene *rpoS* using the ΔΔCt method. For each condition, Ct values were averaged across biological triplicates. ΔCt values were calculated as the difference between the Ct of the target gene and that of rpoS. The ΔΔCt was determined by subtracting the average ΔCt of the control group from the treated group. Fold change in gene expression was calculated using the formula 2^–ΔΔCt.

### Screening of drug library

A custom library of 200,624 unique, non-proprietary compounds custom assembled at Southern Research from various commercial vendors (ChemBridge, Enamine, ChemDiV, Life Sciences, Tripos) was screened in a high-throughput campaign. The compounds met the criteria for lead-like molecules to serve as starting points for a drug discovery effort (molecular weight < =500; heteroatom count < =10; rotatable bonds < = 8; number aromatic rings < =4; ALog P < = 6; molecular polar surface area < =200; H-bond acceptors < 10; H-bond donors < 5). Stock concentrations of library compounds (10 mM dissolved in 100% DMSO) were screened at 10 μM in the presence of 0.6% DMSO, with each compound being tested in duplicate to ensure reproducibility and accuracy. Positive hits, i.e., blocking transparency from developing in the ODS activity assay, from the initial screen were further analyzed through a 10-point dose-response assay to determine their IC50 values.

### Structural prediction and ligand docking analysis of OdsA

The OdsA protein sequence from *P. aeruginosa* PAO1 was retrieved from the Pseudomonas Genome Database (Sequence View: odsA, *P. aeruginosa* MPAO1) [30]. Predicted 3D structural models of OdsA in complex with a heme-dimer were generated in PDB format using the AlphaFold 3 server [31]. The 3D structure of oleic acid was obtained from the PubChem database [32] while 3D models of the top high-throughput screened compounds, AB012 and AB009, were generated using Avogadro (version 1.1.1) [33]. Molecular docking was conducted using the top-ranked AlphaFold model of OdsA and all ligands via Dockey [34] integrated with AutoDock Vina 4 [35]. Protein–ligand complexes were visualized using UCSF ChimeraX [36]. Additionally, the best-docked poses were further analyzed and visualized for key interactions using the Protein–Ligand Interaction Profiler (PLIP) [37].

### Trans-well assay

Trans-well experiments were performed as described previously [38]. Initially, 5.0 × 10^5 murine cardiac vascular endothelial cells (MCEC) were seeded onto Trans-well permeable inserts (12 mm diameter, 3-μm pore size; Costar) in 12-well plates and cultured for a minimum of 48 hours at 37°C with 5% CO2. Subsequently, 5.0 × 10^5 colony-forming units (cfu) of *P. aeruginosa* were introduced to the cells, followed by centrifugation at 500 × g for 5 minutes and incubation for 30 minutes at 37°C with 5% CO2. Following this, the inserts were washed thrice with prewarmed phosphate-buffered saline (PBS) and transferred to new plates, followed by incubation for 1 hour in Dulbecco’s Modified Eagle Medium (DMEM). The translocated bacteria were quantified by enumerating the colony-forming units (CFU) recovered in the lower chamber.

### Quantification of biofilm formation

Biofilm assays were conducted in accordance with the O’Toole protocol [39]. Initially, *P. aeruginosa* strains were cultured overnight on LB agar plates at 37°C. Bacterial suspensions were then prepared in M63 medium to achieve an OD_600_ = 1. Subsequently, 10 microliters of the bacterial suspension were inoculated into each well of a 96-well microtiter plate containing 200 μl of M63 complete media. When necessary, oleic acid or pure oxylipins were supplemented to the medium at desired concentrations. Biofilms were allowed to develop overnight at 30°C. For quantification of biofilms, the wells were washed twice with 1 × PBS, and then 200 μl of 0.1% crystal violet was added to each well, followed by a 10-minute incubation period. Afterward, the wells were washed three times with 1 × PBS, and the crystal violet-stained biofilm was solubilized with 250 μl of 30% acetic acid. Absorbance was measured at 550 nm to determine biofilm formation.

### Virulence assay in mouse burn and excisional wounds model

Mice infection was conducted following previously established protocols [19]. Briefly, five- to six-week-old BALBc mice (Jackson Labs) were anesthetized using vaporized isoflurane at 2.5% in oxygen. Their dorsal region was prepared by shaving with an electric clipper followed by depilation using depilatory cream. For the burn model, A thermal burn, 2 x 3 cm2, was then induced by pressing a hot aluminum bar at 70 degrees Celsius against the skin for 17 seconds which generate a 9% TBSA burn [20,40], after which intradermal infection was administered by syringe using 100 μl of bacterial suspension containing 1.0 X 10^5 CFU (colony-forming units) of either *P. aeruginosa* PAO1 or the *odsAB* mutant strain. For the excisional wound model mice, using sterile scissors, a 5–6 mm full-thickness excisional wound was created on the dorsum. Subsequent to infection, mice received the analgesic buprenorphine (0.1 mg/kg) to alleviate pain. After 48 h, bacterial burden in the skin, liver, and spleen was evaluated. This involved animal sacrifice, collection of tissues, and homogenizing the respective organs and plating serial dilutions onto LB agar plates for quantification.

### *P. aeruginosa* imaging inside mice skin

Mice infected with 1.0 X 10^5 colony-forming units (cfu) of PAO1 or *ΔodsAB* constitutively expressing GFP were euthanized 24 hours post-infection, and a skin biopsy was embedded in Optimal Cutting Temperature Compound (Tissue-Tek, 4583) and frozen until analysis. Visualization of bacteria within the mice skin was performed using the Leica LMD 6 and Nikon Eclipse Ti microscope. Bacterial penetration of skin was determined by measuring the distance of the florescence bacteria from the apical surface of the skin.

### Treatment of burn wound infection with an oxylipin synthase inhibitor

One-hour post-infection, mice were subjected to intradermal treatment, i.e., injection, with either 100 μl of a 10 micromolar solution of AB012 or 100 μl of phosphate-buffered saline for control purposes. Following treatment, mice were monitored daily for symptoms and mortality over a span of 10 days. Alternatively, mice were euthanized at either 24 or 48 hours post-infection to assess bacterial load in the skin, liver, and spleen.

### OdsA expression and mice immunization

The OdsA gene was amplified using the PAO1 chromosome as a template with specific primers. The resulting fragment was then cloned into the PET23a expression vector, incorporating a His tag. This construct was subsequently transformed into *E. coli* BL21 (DE3) for protein expression. Upon reaching an OD600 of 0.4 to 0.6, cultures were induced using 1 mM isopropyl-β-d-thiogalactopyranoside (IPTG) for 4 hours at 37°C on a shaker. Bacterial cells were harvested by centrifugation at 4,000 × g for 15 minutes. The resulting cell pellets were resuspended in buffer A (50 mM Tris-HCl [pH 7.5] and 150 mM NaCl) containing 1 mM phenylmethylsulfonyl fluoride (PMSF), a serine protease inhibitor, and then sonicated at 35% amplitude (2 s on/2 s off) for 30 minutes on ice for lysis. Subsequently, the lysate was centrifuged at 12,000 × g for 30 minutes at 4°C. The overexpressed protein present in the supernatant was purified using a cobalt resin column following the manufacturer’s instructions for His tag purification. For immunization studies, mice were subcutaneously administered 0.1 mg of OdsA in Freud’s completed adjuvant on day 1, followed by a booster of 0.05 mg of the protein in Freud’s incomplete adjuvant administered 14 days later. Peripheral blood samples for serum collection were obtained one-week post-booster via retro-orbital bleeding of anesthetized mice just before euthanasia.

### Detection of oxylipins in the skin of *P. aeruginosa* infected mice

Groups of three mice were subjected to thermal burns and subsequently infected either with PAO1 or a *odsAB* deficient mutant. After 24 hours, the mice were euthanized, and their skin was homogenized using an Omni THQ homogenizer equipped with disposable Omni Tips plastic generator probes (OMNI international) in 2 ml of PBS 1 × . The homogenates underwent centrifugation to remove tissue and bacterial debris, following which total fatty acids were extracted according to the method outlined previously (refer to the section titled “Purification of diol synthase-derived oxylipins”). Extracted samples were then subjected to analysis using HPLC/MS (refer to preceding sections for TLC and HPLC/MS analyses) to detect the presence of 10-HOME and 7,10-DiHOME. The identification of oxylipins was carried out utilizing the Multiple Reaction Monitoring (MRM) method, with mass transitions m/z 297.3/155.1 for 10-HOME and 313.3/141.1 for 7,10-DiHOME.

### Statistical analysis

The survival data from mice experiments were visualized through Kaplan-Meier plots, and their comparability was assessed via the log-rank (Mantel-Cox) test. Each experimental condition involved a minimum of 6 mice. Subsequent analyses utilized either one-way ANOVA or nonparametric Mann-Whitney test. Statistical computations were conducted using GraphPad Prism 8 software (GraphPad Software, La Jolla, CA).

## Supporting information

S1 Fig A) fluorescent microscopy analysis of tissue sections at 48 h post-infection showed limited *ΔodsAB* burden within skin tissue compared to WT PAO1. B) An *in vitro* trans-well assay was performed to assess the ability of PAO1 at equal inocula to cross a confluent MCEC monolayer in the presence or absence of 1mg/mL of oleic acid, for 1 hours. Statistical analyses were performed using a nonparametric Mann-Whitney test with asterisks denoting significance levels (* for P < 0.05). Six mice were utilized per experimental cohort. Shown are the combined results from at least two experiments, with each dot representing a biological replicate.
(TIFF)

S2 Fig A) OdsA tagged with histidine (His-tag) was cloned into the pet23a vector and overexpressed in *E. coli* strain BL21. Lane a represents the uninduced control, while lane b shows the IPTG-induced samples harvested at 1 h, 2 h, 3 h, and 4 h post-induction. B) OdsA-His was then purified using a His-trap column. C) A western blot performed using serum from mice immunized with 0.1 mg of purified OdsA, specifically detected OdsA in the supernatant of PAO1 but not in that of the *ΔodsAB* strain. D) Control experiment showing that immunization with OdsA did not confer protection against *ΔodsAB* colonization of the skin or subsequent dissemination to the spleen E) and liver F), as indicated by the lack of significant differences in bacterial counts. Statistical analyses were performed using a nonparametric Mann-Whitney test. Six mice were utilized per experimental cohort. Shown are the combined results from at least two experiments, with each dot representing a biological replicate.
(TIFF)

S3 Fig High-throughput screening (HTS) strategy and validation of oxylipin synthesis inhibitors. A) Schematic of the HTS assay based on the differential solubility of oleic acid and its oxylipin products. Oleic acid (10 mM) renders a cloudy suspension that becomes transparent upon enzymatic conversion to oxylipins by a semi-purified fraction of PAO1 oxylipin synthases. The optical density at 600 nm (OD600) decreases with oxylipin production, allowing for visual and quantitative identification of inhibitory compounds. B–C) Chemical structures of two lead inhibitors identified from the screen: B) AB00989558 (AB009), an N,N′-disubstituted urea bearing an α-methylbenzyl and a propargyl group and the i*n vitro* dose-response validation of using the oxylipin synthase preparation C) AB01263211 (AB012), an N-(pyridinyl) amide of a spiro-thiolane/cyclohexane system and the i*n vitro* dose-response validation. D) TLC analysis of reaction mixtures showed that both AB012 and AB009 suppressed the production of 10-HOME and 7,10-DiHOME, indicating inhibition of the ODS pathway.
(TIFF)

S4 Fig A) Best docking poses of OdsA and selected ligands, oleic acid, AB012, and AB009. Oleic acid and AB012 bind within the same pocket of OdsA. In contrast, AB009 interacts outside the catalytic pocket, indicating a potential allosteric binding site. B) Binding interaction distances (<4.0 Å) and key residues contacts are consistent with the predicted roles of each inhibitor. These findings support differential modes of inhibition by AB012 and AB009.
(TIFF)

S1 Table Docking parameters and binding affinity metrics for OdsA-ligand interactions. This table summarizes the computational docking results of OdsA with oleic acid, AB012, and AB009. Binding affinity (kcal/mol), root-mean-square deviation (RMSD), unbound RMSD (uRMSD), and inhibition constants (Ki) are presented alongside several ligand efficiency metrics. These include ligand efficiency (LE), fit quality (FQ), size-independent ligand efficiency (SILE), lipophilic ligand efficiency (LLE), and lipophilic efficiency (LELP). The data provide comparative insight into the binding strength, mode, and potential inhibitory efficiency of each ligand against OdsA.
(TIFF)

S2 Table Predicted molecular interactions between OdsA and AB012. The table summarizes the key hydrophobic contacts and hydrogen bonds between the ligand AB012 and amino acid residues within the OdsA binding pocket. A) Hydrophobic interactions: AB012 forms hydrophobic contacts with multiple residues, including Phe260, Trp272, Ile403, Tyr537, Arg540, and Tyr551, at distances ranging from 3.19 to 3.78 Å. These interactions contribute to ligand stabilization within the binding pocket. B) Hydrogen bonds: Three hydrogen bonds are predicted: two involving Thr392 and one with Tyr614. Thr392 interacts with AB012 both as a donor and an acceptor, while Tyr614 contributes an additional hydrogen bond. These polar interactions support the binding affinity and orientation of AB012 in the catalytic site of OdsA. These combined interactions suggest a strong and specific binding profile for AB012 at the catalytic core of OdsA, consistent with its predicted role as a competitive inhibitor.
(TIFF)

S1 Video Interactions between OdsA and ligands AB012 and AB009. AB012 binds at the catalytic site near one of the heme groups, suggesting competitive inhibition with the natural substrate. In contrast, AB009 interacts near the distal heme group, indicating a potential allosteric binding site.
(MP4)
