# Supplementary figures and images for: The Oxylipin Dependent Quorum Sensing System enhances Pseudomonas aeruginosa dissemination during burn-associated infection
Source: PLoS Pathog. 2026 Jan 20;22(1):e1013885. doi: 10.1371/journal.ppat.1013885 (PMC12844534; doi:10.1371/journal.ppat.1013885)

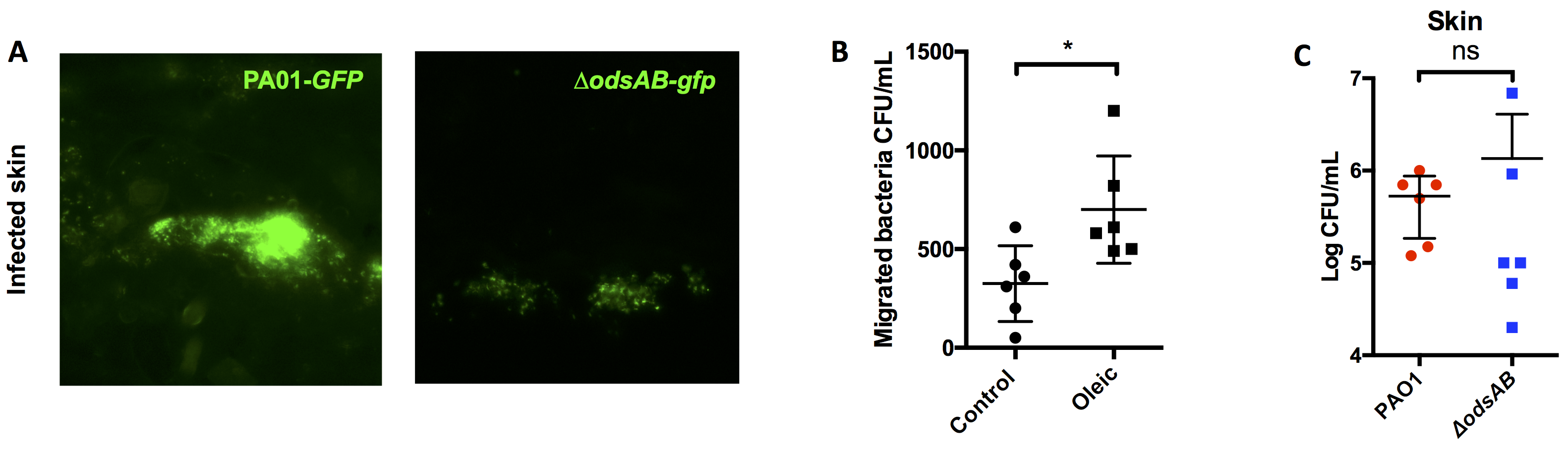

Supplement: S1 Fig — B) An in vitro trans-well assay was performed to assess the ability of PAO1 at equal inocula to cross a confluent MCEC monolayer in the presence or absence of 1mg/mL of oleic acid, for 1 hours. Statistical analyses were performed using a nonparametric Mann-Whitney test with asterisks denoting significance levels (* for P < 0.05). Six mice were utilized per experimental cohort. Shown are the combined results from at least two experiments, with each dot representing a biological replicate. (TIFF) [file ppat.1013885.s001.tiff]

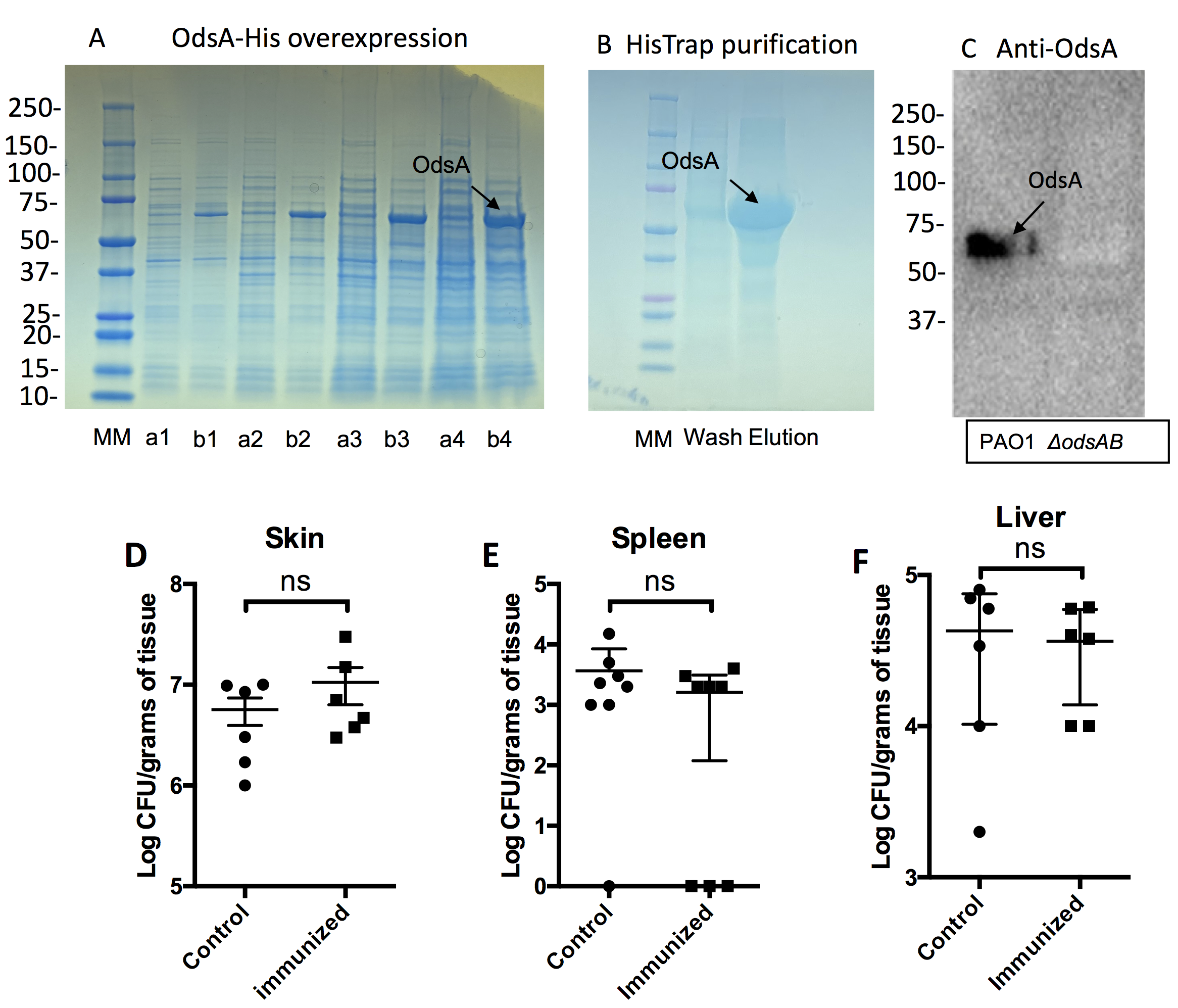

Supplement: S2 Fig — Lane a represents the uninduced control, while lane b shows the IPTG-induced samples harvested at 1 h, 2 h, 3 h, and 4 h post-induction. B) OdsA-His was then purified using a His-trap column. C) A western blot performed using serum from mice immunized with 0.1 mg of purified OdsA, specifically detected OdsA in the supernatant of PAO1 but not in that of the ΔodsAB strain. D) Control experiment showing that immunization with OdsA did not confer protection against ΔodsAB colonization of the skin or subsequent dissemination to the spleen E) and liver F), as indicated by the lack of significant differences in bacterial counts. Statistical analyses were performed using a nonparametric Mann-Whitney test. Six mice were utilized per experimental cohort. Shown are the combined results from at least two experiments, with each dot representing a biological replicate. (TIFF) [file ppat.1013885.s002.tiff]

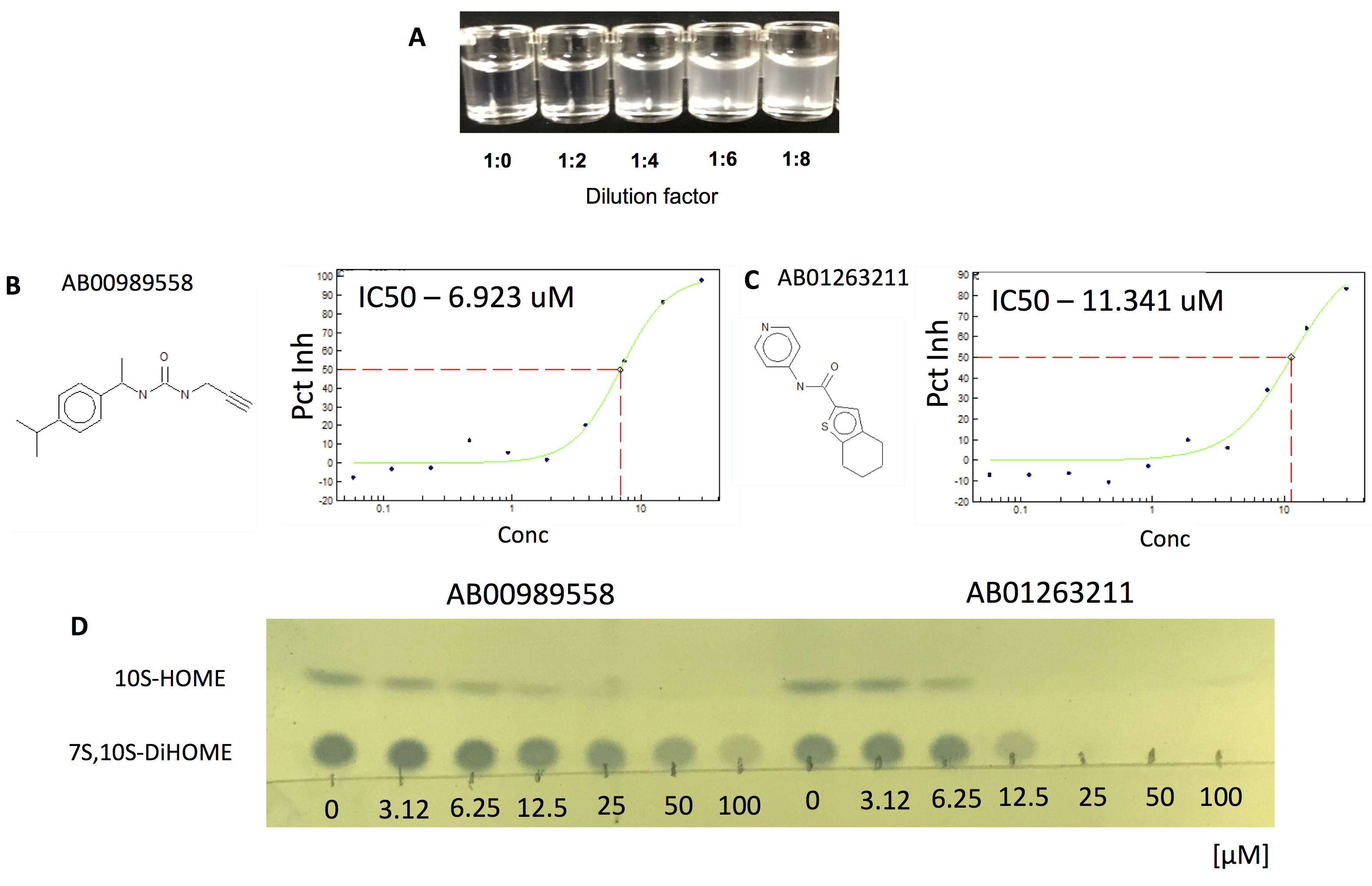

Supplement: S3 Fig — A) Schematic of the HTS assay based on the differential solubility of oleic acid and its oxylipin products. Oleic acid (10 mM) renders a cloudy suspension that becomes transparent upon enzymatic conversion to oxylipins by a semi-purified fraction of PAO1 oxylipin synthases. The optical density at 600 nm (OD600) decreases with oxylipin production, allowing for visual and quantitative identification of inhibitory compounds. B–C) Chemical structures of two lead inhibitors identified from the screen: B) AB00989558 (AB009), an N,N′-disubstituted urea bearing an α-methylbenzyl and a propargyl group and the in vitro dose-response validation of using the oxylipin synthase preparation C) AB01263211 (AB012), an N-(pyridinyl) amide of a spiro-thiolane/cyclohexane system and the in vitro dose-response validation. D) TLC analysis of reaction mixtures showed that both AB012 and AB009 suppressed the production of 10-HOME and 7,10-DiHOME, indicating inhibition of the ODS pathway. (TIFF) [file ppat.1013885.s003.tiff]

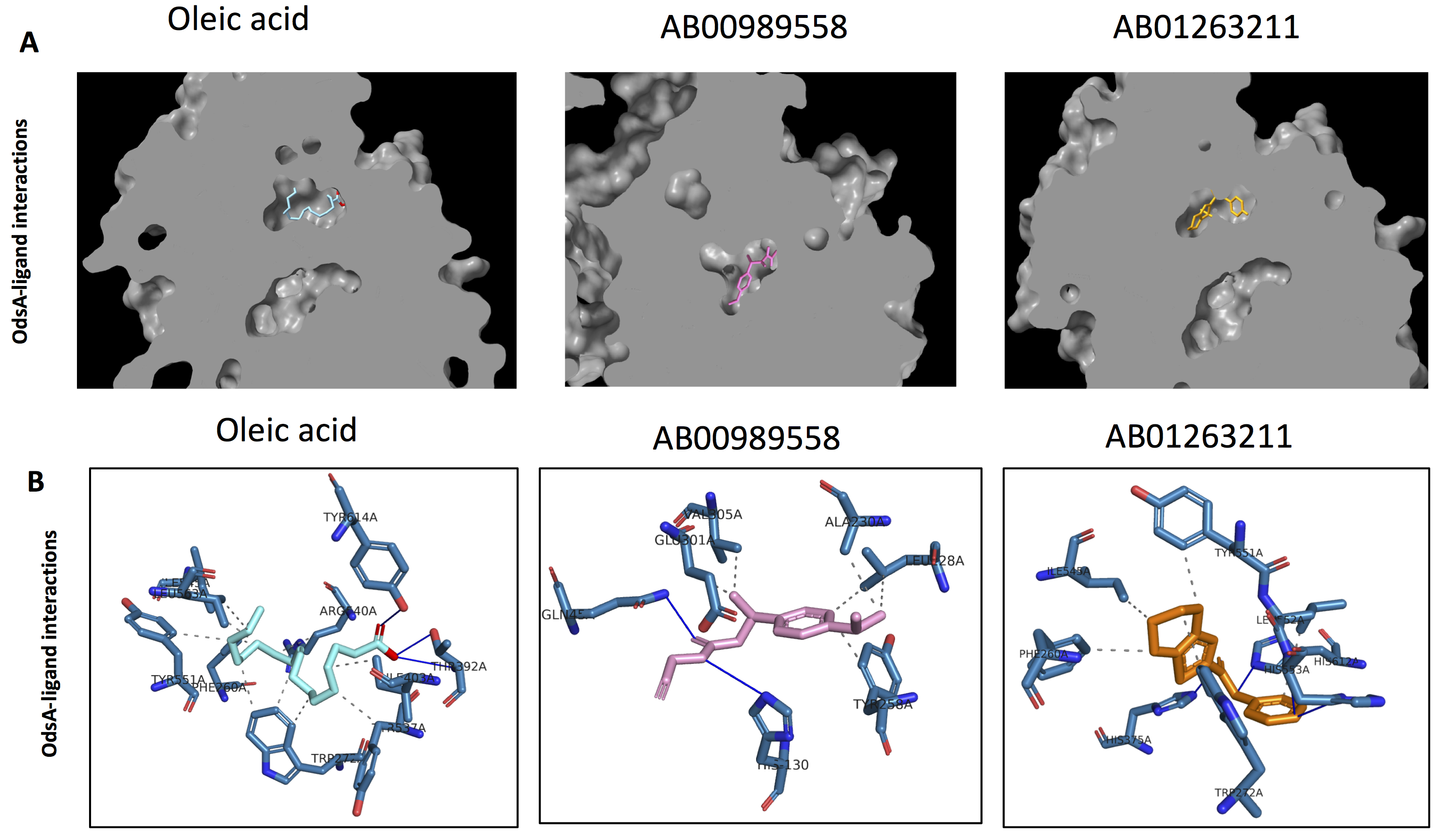

Supplement: S4 Fig — Oleic acid and AB012 bind within the same pocket of OdsA. In contrast, AB009 interacts outside the catalytic pocket, indicating a potential allosteric binding site. B) Binding interaction distances (<4.0 Å) and key residues contacts are consistent with the predicted roles of each inhibitor. These findings support differential modes of inhibition by AB012 and AB009. (TIFF) [file ppat.1013885.s004.tiff]

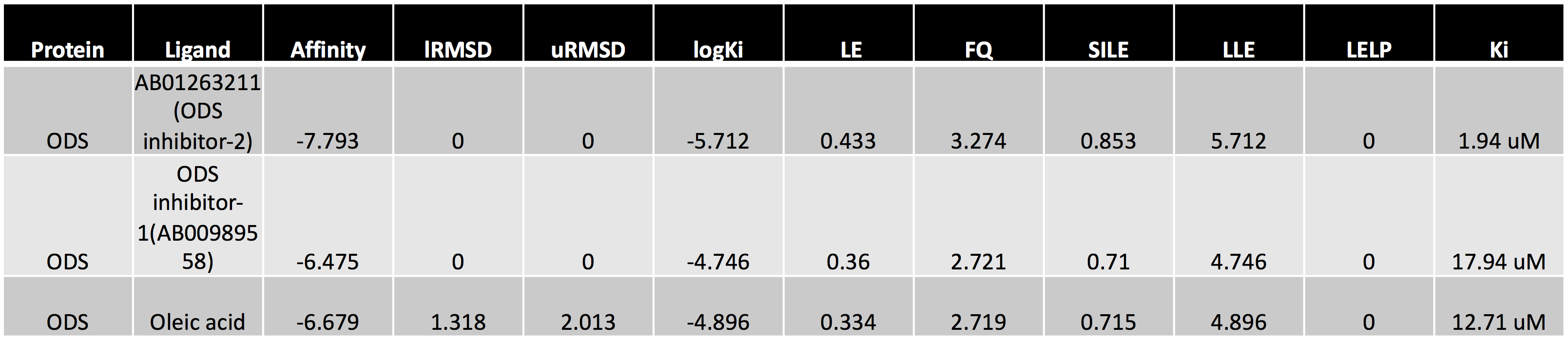

Supplement: S1 Table — This table summarizes the computational docking results of OdsA with oleic acid, AB012, and AB009. Binding affinity (kcal/mol), root-mean-square deviation (RMSD), unbound RMSD (uRMSD), and inhibition constants (Ki) are presented alongside several ligand efficiency metrics. These include ligand efficiency (LE), fit quality (FQ), size-independent ligand efficiency (SILE), lipophilic ligand efficiency (LLE), and lipophilic efficiency (LELP). The data provide comparative insight into the binding strength, mode, and potential inhibitory efficiency of each ligand against OdsA. (TIFF) [file ppat.1013885.s005.tiff]

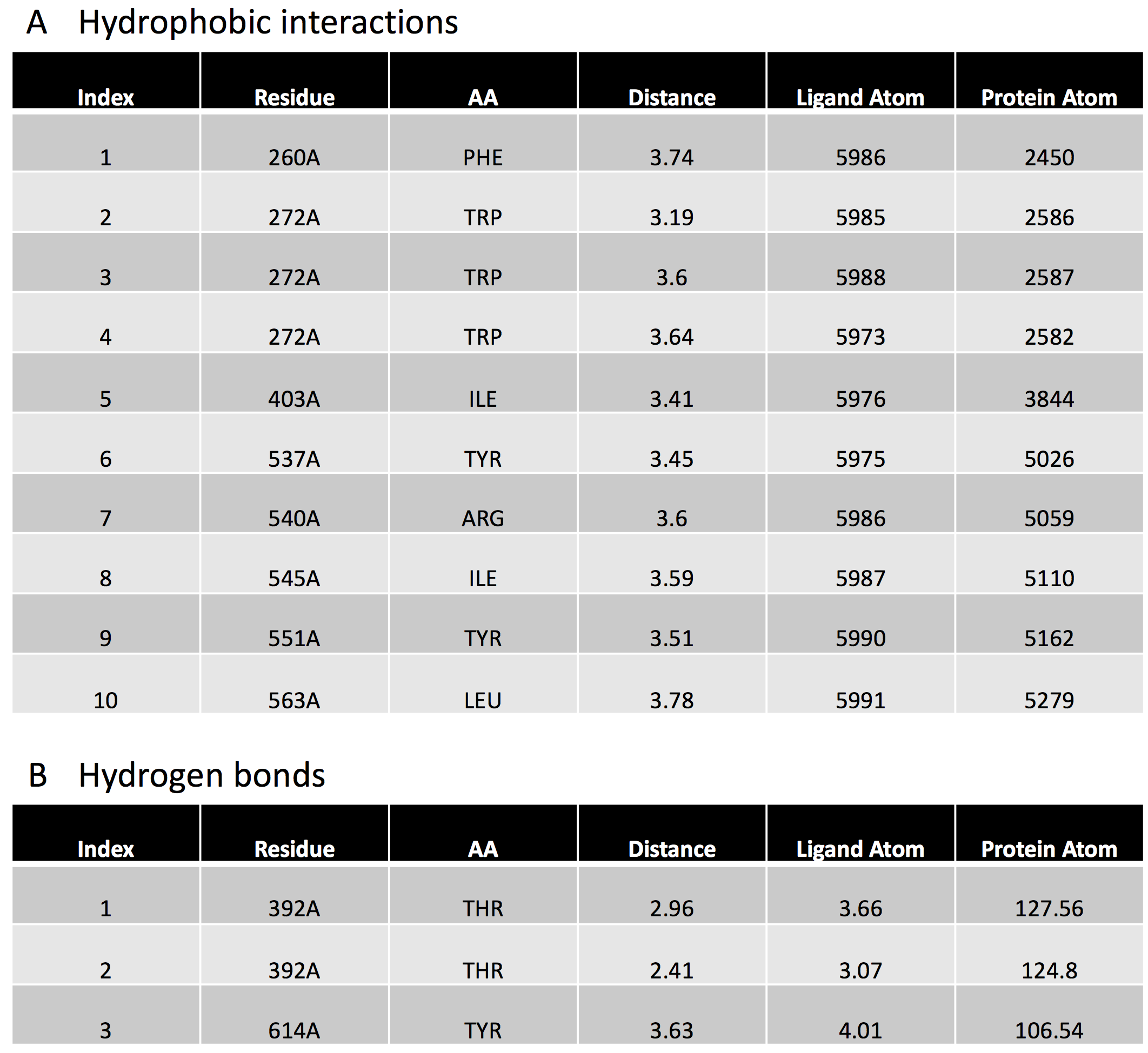

Supplement: S2 Table — The table summarizes the key hydrophobic contacts and hydrogen bonds between the ligand AB012 and amino acid residues within the OdsA binding pocket. A) Hydrophobic interactions: AB012 forms hydrophobic contacts with multiple residues, including Phe260, Trp272, Ile403, Tyr537, Arg540, and Tyr551, at distances ranging from 3.19 to 3.78 Å. These interactions contribute to ligand stabilization within the binding pocket. B) Hydrogen bonds: Three hydrogen bonds are predicted: two involving Thr392 and one with Tyr614. Thr392 interacts with AB012 both as a donor and an acceptor, while Tyr614 contributes an additional hydrogen bond. These polar interactions support the binding affinity and orientation of AB012 in the catalytic site of OdsA. These combined interactions suggest a strong and specific binding profile for AB012 at the catalytic core of OdsA, consistent with its predicted role as a competitive inhibitor. (TIFF) [file ppat.1013885.s006.tiff]
